# Rectified and Salt Concentration Dependent Wetting of Hydrophobic Nanopores

**DOI:** 10.1021/jacs.2c03436

**Published:** 2022-06-21

**Authors:** Jake W. Polster, Fikret Aydin, J. Pedro de Souza, Martin Z. Bazant, Tuan Anh Pham, Zuzanna S. Siwy

**Affiliations:** † Department of Chemistry, University of California, Irvine, Irvine, California 92697, United States; ‡ Quantum Simulations Group and Laboratory for Energy Applications for the Future, Lawrence Livermore National Laboratory, Livermore, California 94551, United States; § Department of Chemical Engineering, Massachusetts Institute of Technology, Cambridge, Massachusetts 02139, United States; ∥ Department of Mathematics, Massachusetts Institute of Technology, Cambridge, Massachusetts 02139, United States; ^⊥^Department of Physics and Astronomy and ^#^Department of Biomedical Engineering, University of California, Irvine, Irvine, California 92697, United States

## Abstract

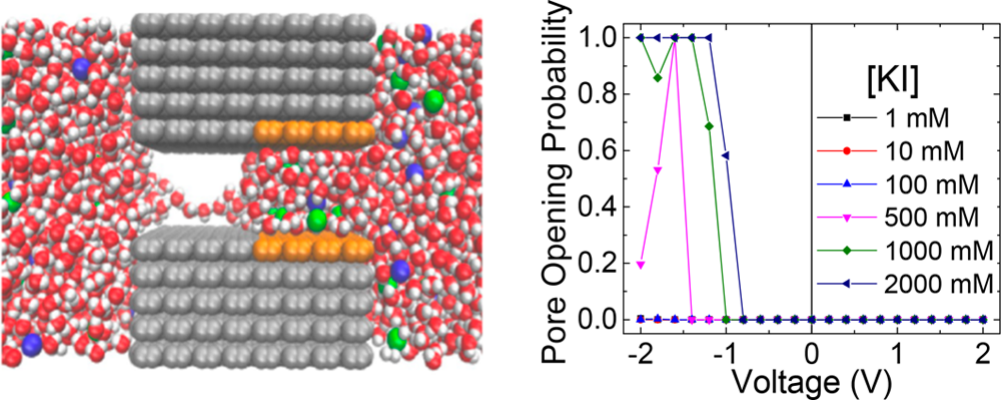

Nanopores lined with hydrophobic groups function as switches for water and all dissolved species, such that transport is allowed only when applying a sufficiently high transmembrane pressure difference or voltage. Here we show a hydrophobic nanopore system whose wetting and ability to transport water and ions is rectified and can be controlled with salt concentration. The nanopore we study contains a junction between a hydrophobic zone and a positively charged hydrophilic zone. The nanopore is closed for transport at low salt concentrations and exhibits finite current only when the concentration reaches a threshold value that is dependent on the pore opening diameter, voltage polarity and magnitude, and type of electrolyte. The smallest nanopore studied here had a 4 nm diameter and did not open for transport in any concentration of KCl or KI examined. A 12 nm nanopore was closed for all KCl solutions but conducted current in KI at concentrations above 100 mM for negative voltages and opened for both voltage polarities at 500 mM KI. Nanopores with a hydrophobic/hydrophilic junction can thus function as diodes, such that one can identify a range of salt concentrations where the pores transport water and ions for only one voltage polarity. Molecular dynamics simulations together with continuum models provided a multiscale explanation of the observed phenomena and linked the salt concentration dependence of wetting with an electrowetting model. Results presented are crucial for designing next-generation chemical and ionic separation devices as well as understanding fundamental properties of hydrophobic interfaces under nanoconfinement.

## Introduction

Hydrophobic interactions under nanoconstriction, such as in nanopores, have been shown to control transport of water and all dissolved species.^1−4^ If a pore wall is lined with hydrophobic groups, in the absence of any external stimuli, such as pressure difference or voltage, the pore will be filled with water vapor even if in contact with a salt solution.^2,5−7^ As the voltage or pressure is gradually increased, the solution will enter the pore only once a threshold stimulus magnitude is reached.^6,8−10^ A hydrophobic nanopore is therefore an ideal valve that stops all transport, including diffusion, in the absence of stimuli or presence of subthreshold magnitude stimulus. Importantly, a hydrophobic nanopore in a wetted state can undergo reversible dewetting once a pressure difference or voltage is decreased or switched off entirely.^6−8^

The possibility of controlling transport in hydrophobic nanopores by electric field is especially exciting. Applying an electric field does not necessitate mechanical strengthening of the pore membrane. Consequently, hydrophobic gating can be achieved even in thin, fragile systems including channels in a cell membrane.^1,8^ In addition, the transmembrane current magnitude is a direct indication of the pore’s wetted or dewetted state and allows one to probe the nanoscale wetting–dewetting transitions occurring within the pore.^5,6^ A dewetted state is observed as negligible current; finite current can only be measured if there is a continuous column of electrolyte along the whole pore length, indicating wetting. Switching between the closed (nearly zero current) and open (finite current) states of a pore is called hydrophobic gating. Wetting of hydrophobic nanopores with an electric field was explained to occur through alignment of water dipoles, which in turns changes water density in the pore and finally leads to wetting.^9,11−13^ Hydrophobic gating with voltage has been demonstrated for biological channels^1^ as well as synthetic polymer^6^ and solid-state nanopores.^5,14^

Recent work demonstrated that hydrophobic gating can also be controlled by placing charged chemical groups in the vicinity of the pore’s hydrophobic zone. In one study, a few charged amino acids present in a model protein nanopore derived from the 5-HT_3_ receptor made the pore’s hydrophobic gating asymmetric with respect to voltage polarity.^8^ When the external voltage had the same polarity as the intrinsic potential difference induced by the charged amino acids, the pore wetting occurred at lower electric field magnitudes compared to the opposite voltage polarity. Another nanopore system where hydrophobic interactions and gating were modulated by the presence of charged groups was created in silicon nitride films.^14^ One entrance of the silicon nitride pore contained highly hydrophobic groups, while the other opening was modified with a positively charged polyelectrolyte. In 1 M KCl, the nanopore was nearly completely closed for positive voltages but opened up for transport at sufficiently high magnitudes of negative voltages.^14^ Voltage polarity dependent wetting was also seen in conically shaped polymer nanopores after modifying their carboxylated surface with long hydrocarbon chains.^15^ The position and density of the hydrophobic modifications were not, however, controlled, and the polymer walls remained overall hydrophilic, with a contact angle below 90°.

The prospect of tuning transport properties of hydrophobic nanopores by placement of surface charges brought another interesting opportunity to control hydrophobic gating with ion concentrations. As the salt concentration decreases, the local electric field that originates from the pore’s surface charges is finite over larger distances from the surface. Consequently, one could hypothesize that less concentrated solutions could promote wetting because surface effects are encompassing more of the pore’s cross-sectional area. The weak dependence of water–air surface tension on salt concentration would also suggest that a smaller external stimulus (such as pressure or electric field) might be required to wet a hydrophobic nanopore in contact with a less concentrated solution compared to the same pore in contact with a more concentrated solution.^10,16^ Interestingly, the conical nanopore system modified with hydrophobic hydrocarbon chains was more likely to wet in higher salt concentrations.^15^ Because the modified polymer walls exhibited contact angle below 90°, the observed salt dependence could not be only attributed to hydrophobic interactions.

In this article we examine the role of bulk ion concentration on wetting of silicon nitride nanopores containing a hydrophobic/hydrophilic junction. Nanopores with opening diameters between 4 and ∼20 nm were fabricated by electron-beam drilling in a transmission electron microscope (TEM).^17,18^ One entrance of the pore was modified with fluorinated alkyl chains while the other opening was modified with amines. The pores were examined in a wide range of concentrations of two salts: KCl and KI. We found that the wetting transition was promoted by an increase in electrolyte concentration. The dependence of the dewetted–wetted transition on ionic concentration was especially clear in solutions of KI. The experimental results are explained by molecular dynamics (MD) simulations that revealed voltage polarity and salt concentration dependent water and ionic concentrations at the hydrophobic/hydrophilic junction. Accumulation of ions in both the hydrophobic and the hydrophilic zones of the nanopore was found to promote wetting. A continuum theory was subsequently applied and utilized ionic concentrations obtained from MD simulations to calculate solid–liquid interfacial energy. A physical model of a hydrophobic system was built that provides analytical equations to predict nanopore wetting as a function of applied voltage and salt concentration.

## Materials and Methods

### Reagents

The following reagents were purchased from the specified company and used as received: potassium chloride (KCl, 99.8%, Fisher Scientific), potassium iodide (KI, ≥99%, Fisher Scientific), tris(hydroxymethyl)aminomethane (Tris, 99.9%, Sigma-Aldrich), (3-aminopropyl)trimethoxysilane (APTMS, 97%, Sigma-Aldrich), 1*H*,1*H*,2*H*,2*H*-perfluorooctyltrichlorosilane (hydrophobic silane, 97%, Alfa Aesar), hydrogen peroxide (H_2_O_2_, 30% w/w, Sigma-Aldrich), and sulfuric acid (H_2_SO_4_, 95–98%, VWR). Milli-Q water (18.2 MΩ) was used for all solutions. Nanopores were drilled in low-stress silicon nitride films (SiN_*x*_, 50 × 50 μm^2^ window, 30 ± 2 nm thick, Norcada).

### Pore Fabrication and Modification

Single nanopores were drilled in silicon nitride films by using a 200 kV electron beam in a JEOL 2100F TEM.^17,18^ Nanopores were fabricated by focusing the electron beam on a single spot for ∼5 min. The silicon nitride films containing the drilled nanopore were then cleaned in piranha solution (3:1, H_2_SO_4_:H_2_O_2_) at 120 °C for 60 min. Once cleaned, nanopores were asymmetrically modified with hydrophilic (bottom) and hydrophobic (top) silanes. First, the film was placed in a homemade polydimethylsiloxane (PDMS) conductivity cell, and the bottom of the cell was filled with a 1% solution of APTMS in ethanol while the top contained pure ethanol (Figure S1a).^19^ The film was exposed to the APTMS solution for 30 min before being rinsed with copious amounts of pure ethanol and subsequently heated at 70 °C for 60 min. Asymmetric modification with APTMS was confirmed by a decrease in transmembrane current and the appearance of ion current rectification.^14^ The film was dried, and any residual salt was removed by submerging first in Milli-Q water followed by ethanol and finally toluene before letting the film air-dry. Once dry, the top of the film was exposed to a 0.2% solution of 1*H*,1*H*,2*H*,2*H*-perfluorooctyltrichlorosilane in toluene for 5 min while the bottom was in contact with pure toluene (Figure S1b). The film was then rinsed in ethanol and heated at 120 °C for 30 min.^14^ Hydrophobic silane modification was confirmed by closed state of the pores at low voltages in KCl solutions.

To estimate the thickness of the modification, we analyzed current–voltage curves of all nanopores before and after the APTMS modification, thus in conditions when the pore could be assumed entirely wet. The values we received ranged from ∼2 nm for the smallest pores (4 nm) to 5 nm for the largest pore (19 nm × 7 nm). The smaller thickness of the attached silane layer in the smaller pore can stem from the hindered access of the reagents to the nanopore.

### Electrochemical Measurements

Ion-current measurements were performed with the patch-clamp amplifiers Axopatch 200B and Digidata 1322A (Molecular Devices, Inc.). The transmembrane voltage was swept from −2 to +2 V in 200 mV steps with a sampling frequency of 10 kHz. Each voltage was held for 50–100 s, with reported values as averages and standard deviations of the time series for the forward sweep, omitting the first and last 5–10 s of each step. To calculate pore opening probability (POP), the total time the pore conducted current was divided by the examined scan time (35 or 85 s, depending on the total scan time).^8^ The threshold current for pore conductance was defined as 6 times the standard deviation of the current at 0 V. Current–time sweeps were analyzed with the event detection program in Clampfit (Molecular Devices, Inc.). All events were inspected, with errors and false positives removed by hand. Discretization of the current–time curves was performed by assigning a 1 or 0 to current values based on the threshold current described above.

Pellet Ag/AgCl electrodes (A-M Systems) were utilized for all electrochemical measurements, with the working and ground electrodes on the hydrophilic (bottom) and hydrophobic (top) sides of the film, respectively. Stock salt solutions (1 and 2 M) were prepared with 10 mM Tris buffer and adjusted to pH 8 before diluting to desired concentration.

### Contact Angle Measurements

Contact angle measurements were performed at room temperature with a homemade imaging setup. Two samples were investigated: unmodified and hydrophobically modified SiN_*x*_ membranes. Contact angle measurements were performed with 2 μL drops of the indicated solution, and contact angles for the two membranes were measured for each solution used in the ion transport experiments. Membranes were washed with Milli-Q water between each measurement. Images were captured on a Nikon D5200 camera outfitted with an Infinity Photo-Optical Model K2 DistaMax long-distance microscope. Contact angle values were calculated by using the ImageJ Contact Angle plugin by M. Brugnara. In addition, water contact angle measurements of SiN_*x*_ membranes that were first modified with APTMS and subsequently modified with three different concentrations of the hydrophobic silane were collected. These contact angle experiments for double-layered SiN_*x*_ membranes were performed on a Kruss DSA30.

### Molecular Dynamics (MD) Simulations

Classical MD simulations were performed by using the LAMMPS simulation package.^20^ Our simulation models consist of a slit pore made of graphene layers and a chloride or iodide aqueous solution with potassium used as the counterion. The pores size is 1.5 nm, which is defined as the distance between the center-of-mass of adjacent graphene layers, and the lateral dimension of the pore is 3.1 nm × 3.3 nm. To mimic the hydrophobic/hydrophilic junction in the experimental system, half of the pore is made hydrophobic by changing the depth of the potential well of Lennard-Jones (LJ) interactions, and the other half is made hydrophilic by adding a positive charge of 0.012*e* to each carbon atom. In this way, ion/water surface chemistry interactions are implicitly captured in the MD simulations. Similar modifications were applied in previous studies; for example, carbon nanotubes were made more hydrophilic by changing the LJ parameters of CNT.^21^ The atoms of the slit pore structure are kept rigid, and their positions are constrained to prevent rigid-body translation of the pore structure during the simulations. We note that the slit opening and length of the pore modeled are an order of magnitude smaller than the pores probed experimentally. This was done to make the modeling tractable and amenable to probing different conditions. We expected these smaller pores to wet in the simulations, as their reduced length was previously shown to facilitate wetting.^22^ Thus, the smaller slit pore could qualitatively describe physical phenomena occurring in a pore containing a hydrophobic/hydrophilic junction that we studied experimentally.

The system was solvated by TIP3P water molecules,^23^ where hydrogen–oxygen bonds were constrained by using the SHAKE algorithm. The ions described by OPLS-AA force fields^24^ were added to obtain a concentration of either 0.1 or 1 M. The long-range electrostatic interactions were solved by using the particle particle-mesh method.^24^ The systems were first energy minimized and then equilibrated under the NPT ensemble by the Berendsen barostat.^25^ The production runs spanning 16 ns were performed under the NVT ensemble by the Nosé–Hoover thermostat,^26^ maintaining the temperature at 298.15 K.

## Results and Discussion

### Voltage Gating of Nanopores with a Hydrophobic/Hydrophilic Junction

**Figure 1:**
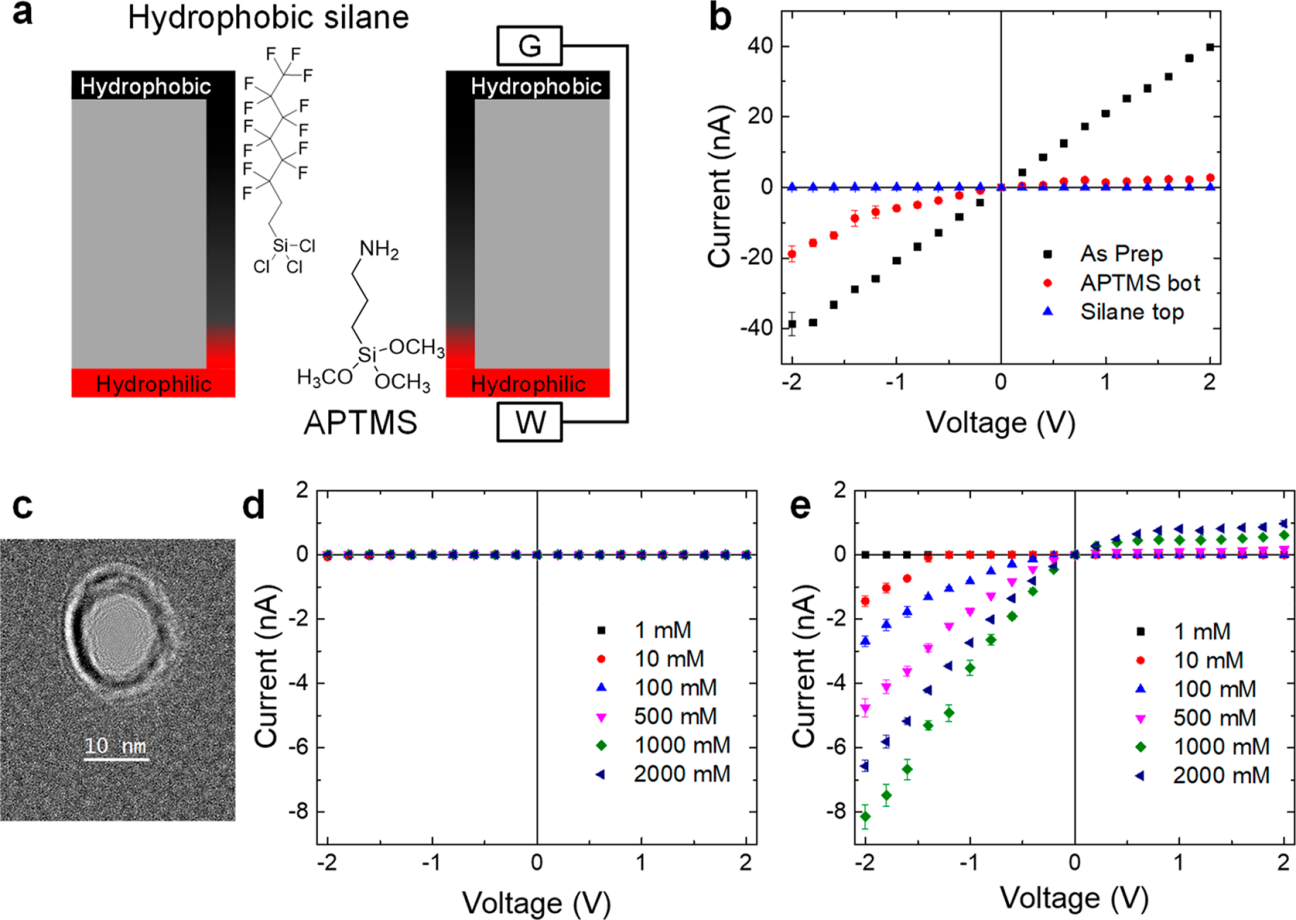
Preparation and performance of a nanopore with a hydrophobic/hydrophilic junction. (a) Nanopore scheme, with electrode (W = working; G = ground) placement as indicated. Nanopores were subjected to two asymmetric modifications, creating a junction between hydrophobic (perfluorooctyl) and hydrophilic (aminopropyl) silanes. Note that the transition between these two zones is expected to be gradual and located close to one pore opening. The junction location was estimated based on contact angle measurements of planar surfaces modified with gradually decreasing concentration of the hydrophobic silane. (b) Current–voltage curves in 1 M KCl for a 12 nm diameter nanopore as prepared (black squares), after APTMS modification (red circles), and after modification with hydrophobic silanes (blue triangles); the pore was prepared in a 30 nm thick SiN_*x*_ chip. (c) TEM image of the 12 nm diameter nanopore as drilled. (d, e) Current–voltage curves in a range of KCl (d) and KI (e) concentrations for the 12 nm pore after the two chemical modifications. The current–voltage curves in panels b, d, and e were obtained by averaging ion current signals recorded at each voltage for 50 or 100 s. Error bars were calculated by standard deviations of ion current signals during recording.

Nanopores used in this article were prepared by electron beam drilling in a transmission electron microscope,^17,18^ and their diameter was measured immediately after fabrication. The nanopores were subsequently subjected to a two-step chemical modification that introduced a junction between a hydrophilic zone and a hydrophobic zone (Figure 1a). Our earlier work showed that only nanopores containing such a junction exhibited hydrophobic gating and could open for ionic transport with applied voltage.^14^ Nanopores whose walls were partly hydrophobic and partly hydrophilic were used here to understand the role of ionic concentration and type of salt on voltage-induced wetting and hydrophobic gating.

To introduce the hydrophobic/hydrophilic junction in a nanopore, a silicon nitride chip with a drilled nanopore was first subjected to asymmetric modification with an aminosilane (APTMS, shown in Figure 1a). To this end, one side of the membrane was in contact with the silane solution, while the other side was in contact with the solvent. This procedure is expected to modify only part of the pore walls since we created two boundary conditions with one pore entrance in contact with the bulk APTMS concentration and the other with zero APTMS concentration (Figure S1a). Assuming a cylindrical shape of the pore, the profile of the silane concentration in the pore is linear,^27^ suggesting that there might be a density gradient of the attached amines along the pore length. Limiting the amination to only a fraction of the walls was, however, assured by the choice of incubation time and APTMS concentration. Previous work on modifications with a similar silane, (3-aminopropyl)triethoxysilane (APTES), showed that, for the same incubation time we used (30 min), a 10 times lower silane concentration led to minimal modification.^28^ We therefore expect that at least 20% of the pore walls will have minimal amine coverage. The presence of a junction between modified and unmodified zones of the pore walls was confirmed by recording rectified current–voltage curves in 1 M KCl at pH 8. The positively charged amino groups and the negatively charged, unmodified silanol groups create asymmetric surface charge distribution within the nanopore, leading to ion current rectification such that current values for one voltage polarity were greater than the opposite polarity (Figure 1b).^14,29^ In our electrode configuration with the working electrode on the APTMS side, currents for negative voltages are larger than currents for positive voltages.

The second and final modification step was aimed at the attachment of hydrophobic groups to the opposite side of the membrane. To this end, 1*H*,1*H*,2*H*,2*H*-perfluorooctyltrichlorosilane was placed in contact with the pore opening that in the amination step was only exposed to the solvent (Figure S1b). Successful attachment of this silane was confirmed by measuring a contact angle of 110° for the modified chip.^14^ Attachment of the hydrophobic silanes is also evidenced by current–voltage recordings because the hydrophobic silane modification would leave the pore in its dewetted state, and the resulting ion current in 1 M KCl would be nearly zero for all voltages (Figure 1b). To probe the depth of the 1*H*,1*H*,2*H*,2*H*-perfluorooctyltrichlorosilane attachment, we modeled the conditions of the modification on a series of planar SiN_*x*_ surfaces. Note that in this modification the side of the membrane that was not modified with amines in the prior modification was now in contact with the hydrophobic silane, while the fully aminated end would be in contact with a much lower concentration of the hydrophobic silane (Figure S1b). Therefore, we modified a series of SiN_*x*_ chips first with APTMS at the same bulk concentration as used before (1%), followed by modification with 1*H*,1*H*,2*H*,2*H*-perfluorooctyltrichlorosilane at the original bulk concentration, and concentrations diminished to 25% and 10% of the bulk. Water contact angle measurements of the chips were then performed. The results indicated that even the lowest concentration considered led to a contact angle of 110°, which is comparable to the surface modified with the maximum concentration. We concluded that the hydrophobic silane could attach to the aminated surface, and the hydrophobic modification extends through most of the pore wall. Consequently, the hydrophobic/hydrophilic junction is expected to be present close to one pore opening (Figure 1a).

Such hydrophilic/hydrophobic nanopores were subsequently tested for their ion transport properties in a wide range of KCl and KI concentrations between 1 and 2000 mM. Ion currents were recorded in the voltage range between −2 and +2 V. The time series of ion current at each voltage were averaged to obtain current–voltage curves. Figure 1d,e summarizes current–voltage curves for the 12 nm diameter nanopore shown in Figure 1c. In KCl this pore remained predominantly closed for all concentrations and voltages and underwent gradual opening in a voltage dependent manner only when subjected to increasing concentrations of KI. The nonconductive state observed in ion-current measurements of KCl indicates that the pore was not entirely filled with liquid water and thus closed.^6,8,14^ On the basis of previous work,^1,6−8,11,14^ we believe that when the pore was closed, a zone of water vapor existed in its cross section, preventing transport of liquid water and ions. On the other hand, recording finite ion current is only possible if there is a continuous column of water along the entire pore length. In 1 mM KI no measurable current was observed for any voltage polarity, suggesting the pore was at least partly dewetted. At 10 mM KI, the pore began opening for ion transport and conducted finite current for negative voltages equal and larger in magnitude than −1.4 V. For 100 mM KI, the pore conducted finite ion current for all negative voltages but remained closed at positive voltages. Only when the KI concentration was increased to 500 mM did the pore become conductive for both positive and negative voltages. Note that for negative voltages currents recorded in 1 M KI are larger than currents in 2 M KI. We believe this effect could stem from a stronger screening of charges in the higher concentration that weakens the enhancement of ionic concentrations within the pore and is responsible for the nonlinear current increase with negative voltage.^30−32,29,33^ The current for positive voltages, on the other hand, follows the expected dependence on salt concentration, with 2 M KI producing the largest current. The difference in the concentration dependence of ion current for negative and positive voltages can be understood through the rectifying properties of this pore. In rectifying nanopores, the voltage polarity that produces lower currents, positive voltages in our case, leads to a depletion zone of ions in the pore.^30,34^ However, with the increase of salt concentration, the surface charges are more screened and more ions reside in the pore, thus preventing the depletion zone from completely developing in higher salt concentration solutions. Consequently, positive currents follow bulk solution conductivity trends.

The differences in ion transport properties for KCl and KI solutions are unique to nanopores that were subjected to two asymmetric modifications: first with APTMS, followed by modification with 1*H*,1*H*,2*H*,2*H*-perfluorooctyltrichlorosilane, as described above. As-prepared nanopores^14^ as well as nanopores entirely modified with APTMS (Figure S2) were open for ionic transport in both KCl and KI and produced nearly identical, linear current–voltage curves in the two salts. These results agree with the salts’ nearly identical bulk conductivities. Moreover, nanopores that were modified symmetrically with the hydrophobic silane did not open for transport with external voltage either in KCl or KI.^14^ The clearly distinct transport properties of the asymmetrically modified nanopores provide additional evidence for the existence of a junction between aminated and hydrophobic zones of the pore walls.

The more conductive nature of the hydrophobic/hydrophilic nanopores in KI versus KCl aqueous solutions agrees with our earlier molecular dynamics results that revealed accumulation of large, polarizable ions, such as iodide, on the hydrophobic pore walls.^14^ The strong influence of the salt concentration on wetting was, however, surprising. As shown in Figure 1, the nanopores were more likely to open for ionic transport in higher salt concentrations than at lower salt concentrations. As surface tension of a water–air interface exhibits a weak increase with the increase of salt concentration,^16^ an analysis based on surface tension alone would actually predict only a weak and possibly opposite effect of ionic strength on pore wetting to what we are reporting here.^16^ In addition, the contact angle of the hydrophobic surface probed here has not shown an obvious dependence on the salt concentration of either KCl or KI (Table S1).

### Analysis of Ion Current Time Series and Hydrophobic Gating

**Figure 2:**
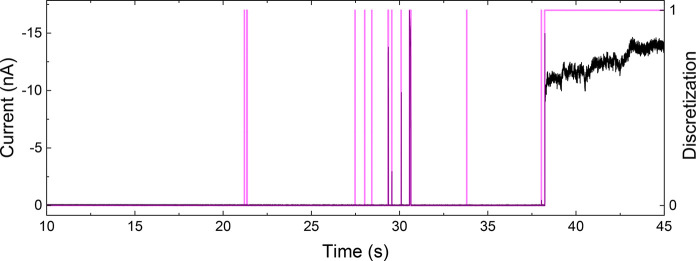
Recording of ion current time series for the pore shown in Figure 3d for 500 mM KI at −2 V. The experimental data displayed in black is discretized into {0, 1} states indicating closed and open states of the pore, shown in magenta. The pore opening probability for this voltage was 0.2.

To visualize the voltage-induced wetting of nanopores and to facilitate comparison of recordings at different salt concentrations, we carefully analyzed the ion current time series. Nanopores studied here had opening diameters below 20 nm and were found to exhibit spontaneous switching between nonconductive and conductive states, even at a constant voltage (see example in Figure 2). We changed the raw current recordings into a dichotomous, two-state signal consisting of level 0, the closed state with no current, and level 1, the open state with finite current.^8^ A nanopore was considered conductive/open for ionic transport if the ion current signal was larger than 6 times the standard deviation of the current signal at 0 V. At each voltage we determined the fraction of the recording a nanopore spent in its conductive, {1}, state, and this fraction became our measure of the pore opening probability (POP).^8^ This analysis allowed us to find the dependence of pore wetting on ionic concentration, applied voltage, and pore diameter.

Note that before the nanopore in Figure 2 reached a long-lasting open state at the end of the recording, there were brief bursts of finite current separated by seconds long periods with zero current. The long duration of the closed state suggests that the air bubbles created in the nanoscopic system can be very stable, in agreement with previous results,^35^ as discussed in the modeling section below. The opening of the nanopore at ∼38 s indicates formation of a long open state, but as shown in Figure S3, the subsequent sweep at −1.8 V started with the pore in a closed state again. These recordings demonstrate that the time scale of the hydrophobic gating spans many orders of magnitude, and more studies are needed to understand the origin of the short and long wetted and dewetted states. As described below, such gating exists right at the threshold voltage and concentration values. This is likely due to hydrophobic hysteresis,^5,14,36^ where both states are metastable.

**Figure 3:**
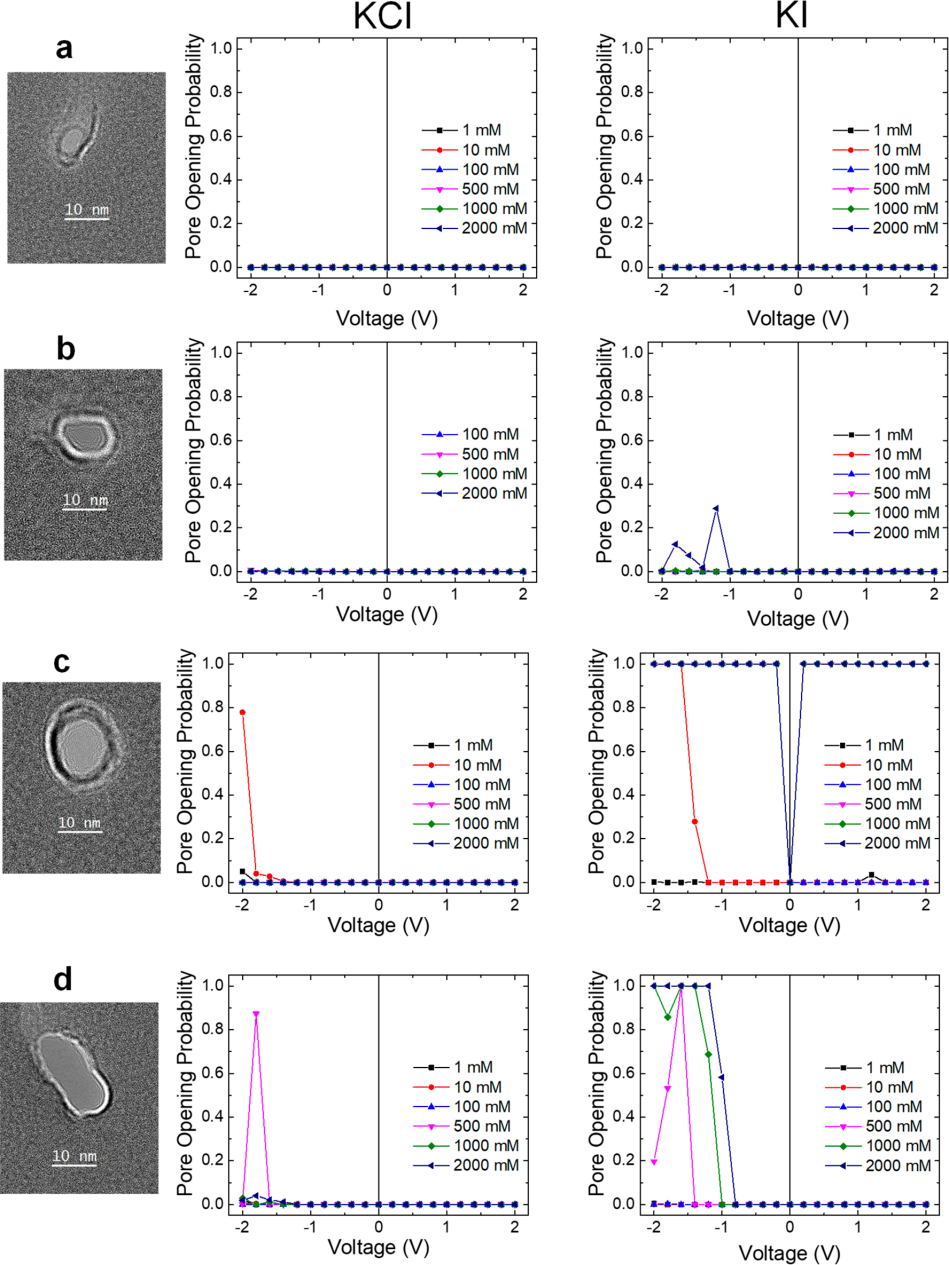
Pore opening probability for nanopores in a wide range of KCl and KI concentrations. (a–d) TEM images of as prepared pores are shown on the left. The middle and right-hand side panels show data for KCl and KI, respectively.

Figure 3 summarizes the pore opening probability of four nanopores with different opening diameters, all below 20 nm, as a function of salt concentration and voltage. The smallest nanopore we examined was 4 nm in diameter and did not open for ionic transport in either KCl or KI for any examined concentrations, as shown in Figure 3a. We suspect that the voltage range of −2 to +2 V was insufficient to induce wetting.^5,6,8^ Another pore had a 6 nm diameter and remained closed in KCl but opened for ionic transport only in 2000 mM KI, the largest examined concentration, at negative voltages (Figure 3b). A POP analysis for the 12 nm diameter pore shown in Figure 1 confirmed its closed state for nearly all concentrations of KCl and gradual opening with the increase of KI concentration (Figure 3c). Note: the 12 nm nanopore conducted finite current for both positive and negative voltages when the KI concentration reached 500 mM. Finally, the nanopore in Figure 3d was not circular and measured 19 × 7 nm^2^. The oblong shape of this pore resulted from a slight drift of the e-beam in the TEM while drilling. In KCl, this pore remained mostly closed, except for 500 and 2000 mM at high negative voltages. In KI, on the other hand, the pore was closed for all voltages at concentrations ≤100 mM and started to conduct current at negative voltages for KI concentrations ≥500 mM. As the concentration increased from 500 to 2000 mM, the pore transported ion current for a wider range of negative voltages, qualitatively following the same trend as the pores in Figure 3b,c. Higher KI concentrations lowered the threshold voltages required to open the pore, decreasing from −1.4 V for 500 mM to −0.8 V for 2000 mM. The 19 × 7 nm^2^ oblong nanopore did not open for ion transport at positive voltages, most likely due to one of its axes measuring below 10 nm. Note: the sub-10 nm pores in Figure 3a,b indeed did not conduct current at positive voltages for either electrolyte. The presence of the larger axis, however, made the pores conductive in lower magnitudes of negative voltages compared to sub-10 nm circular nanopores. Thus, transport properties of oblong nanopores are determined by both axes.

**Figure 4:**
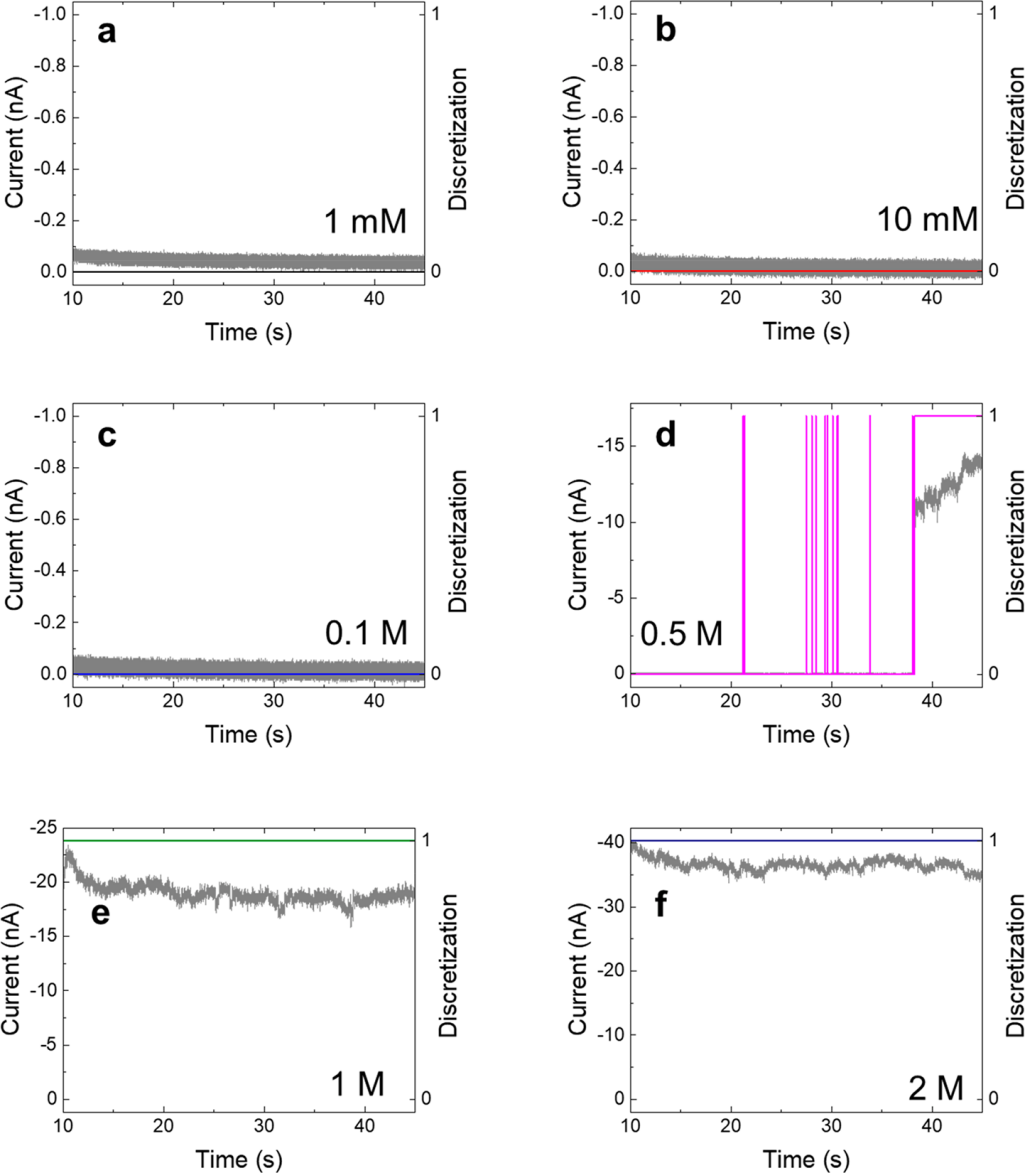
Time-resolved recordings of ion current for the nanopore shown in Figure 3d at −2 V for six different KI concentrations, as indicated in panels a–f.

Similar to the existence of a pore diameter and voltage threshold, there also seems to be a concentration threshold at which a pore begins to open. For nanopores with an opening above 10 nm (Figure 3c,d), the threshold concentration was between 10 and 500 mM. Abrupt current openings and closings, seen in Figures 2 and 4d, were observed for intermediate concentrations and voltages, where the pore underwent intermittent wetting and dewetting. These conditions yielded pore opening probabilities in the 25–75% range.

The ability to control gating with ionic concentrations is especially evident when examining the dichotomous current–time graphs recorded for the same voltage but different salt concentrations (Figure 4). All current–time graphs in Figure 4 were recorded at −2 V in KI for the nanopore shown in Figures 2 and 3d. For 1–100 mM, the current never reached past the threshold required to be conductive (Figure 4a–c). At 500 mM (Figure 4d), there were a few short bursts of current followed by a continuous opening toward the end of the sweep. Finally, recordings at 1000 and 2000 mM KI (Figure 4e,f) revealed continuous conductance of the oblong nanopore (Figure 3d). These six sweeps show that the salt concentration plays an important role in the pore wetting, with intermediate concentrations displaying unstable conductance.

Another striking feature of the recordings shown in Figures 1 and 3 is the ability for nanopores with a hydrophobic/hydrophilic junction to function as a diode for water and all dissolved ions, such that transport is allowed mostly at negative voltages. A similar asymmetric voltage response was observed earlier only in KCl with nanopores that were few tens of nanometers in diameter and contained such a hydrophobic/hydrophilic junction.^14^ The voltage polarity (negative) that promoted pore opening was determined by the migration direction of counterions (anions) in the positively charged hydrophilic zone. Pores would conduct ionic transport for the voltage polarity that sourced counterions from the reservoir in contact with the hydrophilic zone. In the system presented here, for negative voltages that facilitate wetting, anions are indeed sourced from the pore entrance decorated with APTMS and transported toward the hydrophobic entrance. In addition, the hydrophobic/hydrophilic nanopores shown here are conductive only after a threshold voltage and threshold concentration of KCl or KI was reached. The two stimuli–voltage and electrolyte concentration–work synergistically. As the salt concentration increased, the pores exhibited lower threshold wetting voltages for negative polarity and were more likely to open at positive voltages. All these observations provide strong evidence that salt concentration and pore opening probability are directly related.

The possibility of controlling the wetting–dewetting transition in nanopores with salt concentration, ion type, and applied voltage is modeled below by molecular dynamics simulations as well as by using a mechanistic approach involving the existence of air bubbles and electrowetting. The molecular dynamics approach provides an atomistic insight into pore wetting and dewetting at different experimental conditions, while the continuum model (*infra vide*) considers the energy associated with the ionic adsorption and charging. The model predicts that wetting is indeed facilitated by the increase of ionic concentration that leads to adsorption of ions to the surface and lowering of the effective solid–liquid interfacial tension.

### Molecular Dynamics Simulations of Nanopores with Hydrophobic/Hydrophilic Junction

**Figure 5:**
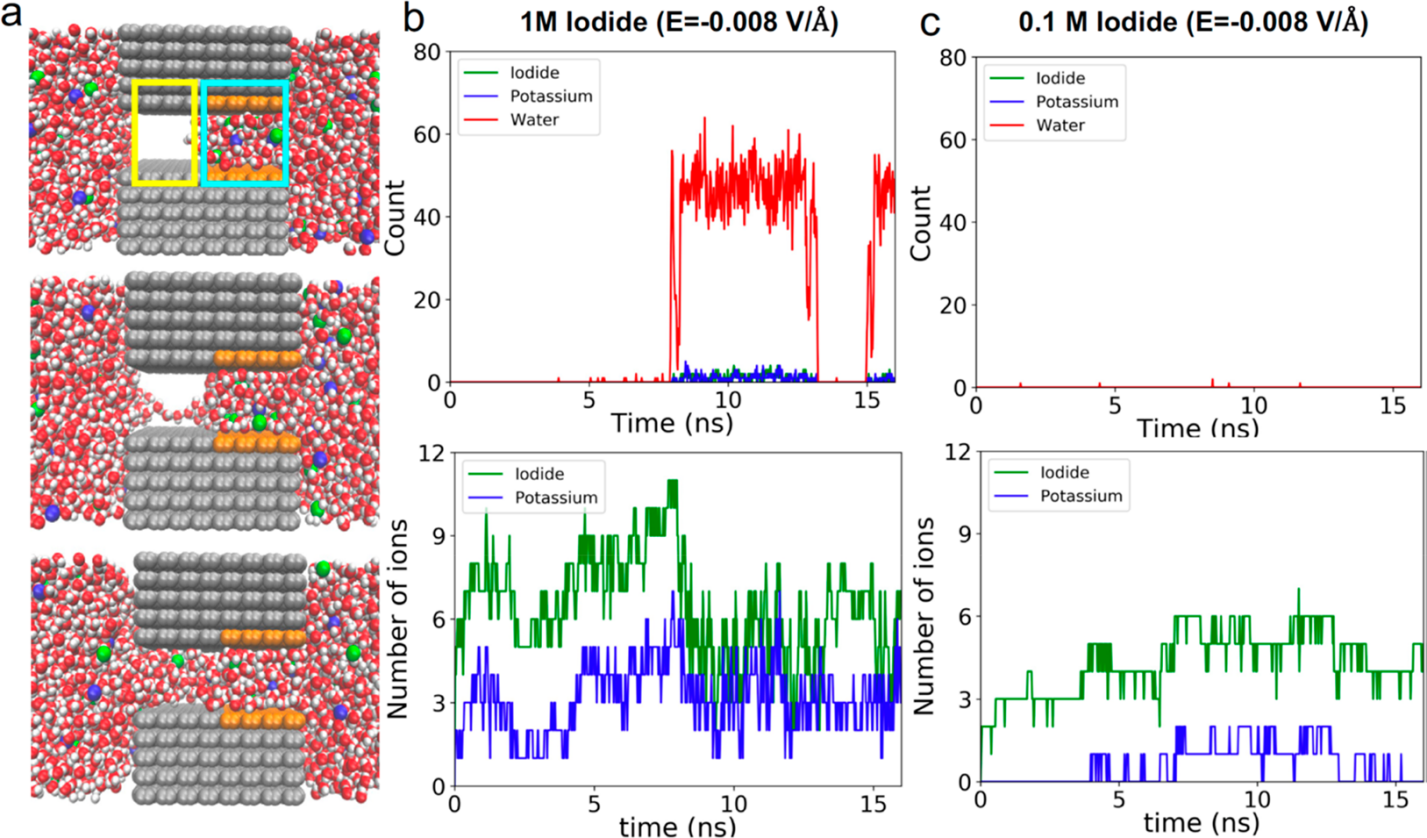
Molecular dynamic simulation results for a hydrophobic/hydrophilic nanopore model. (a) Simulation snapshots showing different stages of pore wetting in 1 M KI salt solution at a negative electric field of 0.008 V/Å. Areas encircled by yellow and cyan lines correspond to hydrophobic and hydrophilic regions of the pore, respectively. (b, c) Number of water molecules, potassium, and iodide ions in the hydrophobic (top panels) and hydrophilic (bottom panels) regions of the pore as a function of time for (b) 1 M KI and *E* = −0.008 V/Å and (c) 0.1 M KI and *E* = −0.008 V/Å.

Molecular dynamics simulations of a model system were performed to provide insights into the mechanism for how ion concentration and applied potentials promote wetting of nanopores with a hydrophobic/hydrophilic junction. We considered a 1.5 nm wide and 3.1 nm long slit pore made of hydrophobic graphene layers. Half remained uncharged and hydrophobic, and the other were assigned net positive charge (Figure 5a). This model system allowed us to probe the importance of the junction between two zones with dissimilar chemical properties without considering its exact position in the pore. To explore the effect of applied voltages on nanopore wetting, electric fields with different magnitudes and polarities were applied across the model nanopore. We considered electric fields of 0.005 and 0.008 V/Å, as they are comparable to experimental conditions. We note that 0.005 V/Å is equivalent to a 1.4 V potential across a 30 nm thick film, which was often insufficient to lead to pore wetting (Figure 3). On the other hand, 0.008 V/Å represents the maximum voltage employed experimentally (2 V). Herein, the two electric fields will be called low and high, respectively. The simulations were performed in 1 and 0.1 M solutions of KI and KCl.

We first considered the hydrophobic region of the model nanopore that determines when the transport of ions can occur. Our simulations showed that the nanopore was not wet at the low electric field, regardless of the ion type, electric field polarity, or salt concentration. On the other hand, at the high electric field (0.008 V/Å) and 1 M KI, the pore was filled with water and became conductive only at negative potentials; the same nanopore remained closed at 1 M KCl (Figure S4 and Figure 5b top panel). The salt dependence could be explained by the weaker solvation shell of iodide ions that enable their accumulation on the hydrophobic surface, as reported by us before,^14^ as well as their accumulation near water–vacuum interface (Table S2). The simulations also reproduced experimentally observed dependence of the nanopore wetting on salt concentration. At the high electric field, when the salt concentration was lowered to 0.1 M, the nanopore did not open for ionic transport in either salt (Figure 5c, top panel, and Figure S4). Overall, these observations are consistent with experimental findings shown in Figures 1 and 3. Furthermore, the analysis provides a more detailed understanding of the wetting process; for example, the simulations indicate that the hydrophobic region is first filled with water molecules, followed by the influx of ions that occurs within a time scale of a fraction of a nanosecond (Figure 5a, middle panel, and Figure 5b, top panel). Similar behavior has been observed for biological hydrophobic pores.^11^

To further understand the effect of ion concentration on nanopore wetting, we also calculated the number of water molecules and ions in the hydrophilic region of the pore (Figure 5b,c, lower panels, and Figures S5 and S6). As expected, the hydrophilic zone is filled with water in all conditions (Figure S6), confirming that the transport properties of the nanopore are dominated by the state of the hydrophobic zone. The modeling also confirmed anion selectivity of this region, especially pronounced in 0.1 M KCl and 0.1 M KI and even in 1 M in conditions when the pore was not wet (Figure S5). Only at −0.008 V/Å, 1 M KI after ∼8 ns when the hydrophobic zone was filled with water (Figure 5b, lower panel) did the concentrations of potassium and iodide became nearly identical. These results allowed us to conclude that concentrations of ions in hydrophobic and hydrophilic regions are coupled, and the pore wetting is also facilitated by the increased ionic concentrations in the hydrophilic region.

Finally, we investigated the stages of pore wetting by examining snapshots from the simulations (Figure 5a). First, wetting was initiated by a short string of water molecules instantaneously emerging from the bulk solution. This was followed by connecting two such strings of water molecules that were initiated from both sides of the hydrophobic region. The connected string of water molecules was found to grow with time to finally fill up the entire hydrophobic region of the pore. We also looked at the molecular details of the inverse process of dewetting observed in Figure 5b at ∼13 ns. The region filled with water was found to shrink with time, and eventually strings of water molecules were completely disconnected so that the pore became fully dewetted (Figure S7).

In summary, the molecular dynamics simulations revealed voltage- and concentration-controlled wetting of a nanopore with a hydrophobic/hydrophilic junction. The large number of ions present in the hydrophilic region at high bulk concentrations likely causes disruption of the hydrogen bond network and facilitates pore wetting by enabling formation of water strings from the bulk solution. The simulations also suggest that formation of the water strings is further facilitated by the weaker solvation shell of iodide ions, as the water–water interactions are destabilized due to the accumulation of the iodide ions at the hydrophobic surface. These results provide explanation for the experimentally observed anion dependent wetting of the pores.

The modeling also confirmed that the hydrophobic/hydrophilic junction is crucial for breaking symmetry of the system and the diode-like behavior of these pores. In an entirely hydrophobic pore, ionic concentrations are not expected to be dependent on the pore axial position or voltage polarity.^14^ Thus, the diode-like behavior and controllable gating are dependent on the presence of a hydrophobic/hydrophilic junction. The importance of contrasting hydrophobic/hydrophilic properties of two pore entrances for electric field induced wetting was already suggested by earlier studies with a protein nanopore derived from the 5-HT_3_ receptor.^8^ The biological structure contained charges near one opening, which did not induce as such a striking rectifying behavior as in the nanopores presented here but was sufficient to result in voltage polarity dependent wetting. For the next step, we developed a continuum model to provide an analytical formula describing the influence of ionic concentrations and voltage on wetting.

### Description of Nanopore Wetting Using an Electrowetting Model

The position dependent ionic concentrations found through MD simulations shown here and in ref (14) were subsequently used to provide a continuum model of the wetting process as well as analytical equations that predict wetting conditions. To qualitatively explain the dependence of nanopore wettability on salt concentration and voltage, we applied a continuum model of wettability including ionic surface adsorption, as described in ref (37) based on the thermodynamic analysis of double-layer charging.^38^ Here, we compute the energy associated with the ionic adsorption and charging to determine the change in effective solid–liquid interfacial tension.

While the interfacial tension is central in determining the wettability for macroscopic bubbles, bubbles at the nanoscale, such as those one might expect to exist encompassing and within the nanopores in our study, exhibit much more complex nanoscale physics,^35^ including contact line pinning, dynamic equilibrium, and surface heterogeneities. Therefore, we do not expect this model to give quantitatively exact predictions of the bubble dynamics, but rather we use this model as a guide to understand the possible physics at play in the hydrophobic/hydrophilic system.

**Figure 6:**
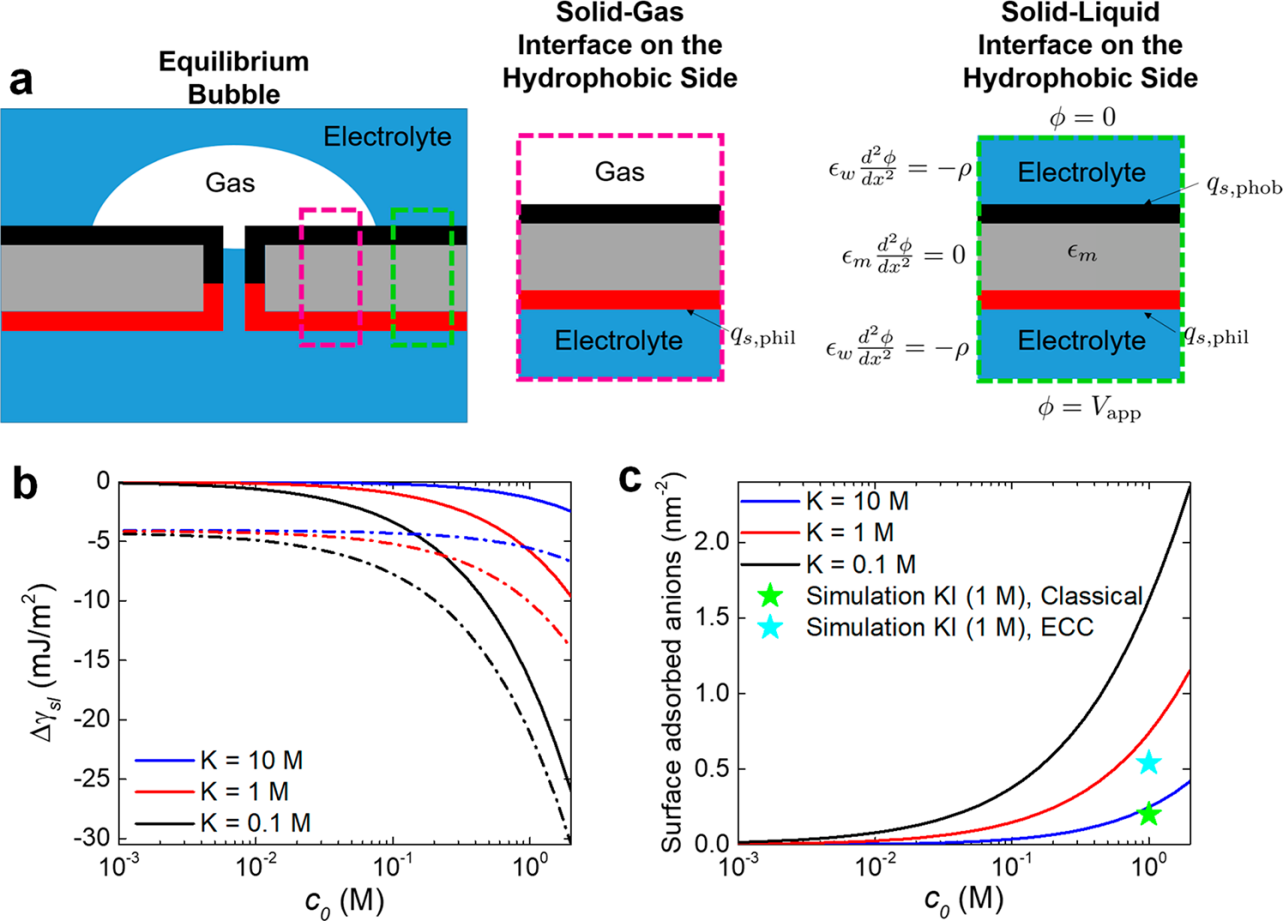
Continuum model of nanopore wetting. (a) Schematic of the continuum model, where the electrostatic potential is solved for as a function of the applied potential difference, and the bulk ionic concentration. (b) Change in the effective solid–liquid interfacial energy, Δγ_sl_, as a function of the ionic concentration for varying anionic adsorption equilibrium constant, *K*, and applied voltages, *V*_app_. The solid lines correspond to an applied voltage of 0, and the dashed dotted lines correspond to an applied voltage of +2 V. The change in interfacial energy is not strongly dependent on the sign of the applied voltage since local concentration polarization within the pore domain is not captured by the model. (c) Surface charge density of the hydrophobic side as a function of the salt concentration for varying anionic adsorption equilibrium constant, *K*. Because the membrane is only weakly polarizable, the surface charge density is only a weak function of the applied potential (not shown). Here, the stars are shown for two different simulations of KI solutions at 1 M to indicate the expected value of adsorption coefficient. Fixed Parameters: *N* = 5 nm^–2^, ϵ_m_ = 7ϵ_0_, ϵ_w_ = 80ϵ_0_, *L* = 30 nm, *T* = 300 K, and *q*_s,phil_ = 0.06 e nm^–2^.

Our wettability model is based on the approach presented in ref (37) which we extended to include the polarization of the membrane domain that is coupled to the double layers in the solution domains. Here, we solve for the electrostatic potential in the membrane and in the double layers at the membrane interfaces^39^ and then use that profile to compute the effective surface energy. The formulation assumes anion adsorption on the hydrophobic side of the membrane that varies as a function of the local anion concentration, while the surface charge on the hydrophilic side is assumed to be positive and constant. Note that the adsorption component allows us to take into account the differences in local concentrations of chloride and iodide ions at the hydrophobic surface/liquid interface, revealed by MD simulations.^14^ To avoid the geometric complexity of the pore and its opening within the membrane, we focus our model on the wettability further away from the pore, where we can safely assume that the potential varies only in the normal direction to the membrane surface. Such an assumption would be relevant for large bubbles (large relative to the pore length scale) existing far from the pore entrance. Even though such a simplified geometry misses the detailed potential profile near the pore mouth and bubble interfaces, as well as the profile around the bubble corners,^40^ it provides an intuitive model to capture the role of ionic concentrations and voltage in wetting. A schematic of the continuum model is included in Figure 6a. Details of the theoretical basis and solution of the continuum model are presented in the Supporting Information.

The key parameters of the model include the anionic adsorption equilibrium constant, *K*, the thickness of the membrane, *L*, the Debye length, λ_D_, the salt concentration, *c*_0_, the applied electrostatic potential difference, *V*_app_, the number density of surface sites for anion adsorption, *N*, and the membrane and solution permittivities, ϵ_m_ and ϵ_w_, respectively. As the anionic adsorption equilibrium constant decreases or the ionic concentration increases, the adsorption of ions to the surface is enhanced, leading to the increase in surface charge density. The higher surface adsorption gives two contributions that make wetting more favorable: (i) the electrostatic energy stored in the diffuse double layers and (ii) the energy of surface anion adsorption. As the applied voltage across the membrane increases, charge is stored across the polarizable membrane, providing a favorable contribution to wetting. Figure 6b shows the change in the effective surface energy as a function of the ionic concentration for different anion adsorption equilibrium constants and different applied voltages. The full set of nonlinear formulas for solid–liquid interfacial energy, Δγ_sl_, is shown in the Supporting Information.

While the full theory requires the solution of a coupled set of nonlinear equations, we can also derive analytical, explicit solutions to the energy for the double layer assuming small surface potentials and a weakly polarizable membrane. The small surface potential relative to the thermal voltage leads to a constant capacitance in the double layers. The weakly polarizable membrane assumption, applicable due to the large thickness of the membrane relative to the Debye length and the membrane’s low dielectric constant relative to the solution, allows us to decouple the contributions to the energy from the membrane and the independent double layers. The various contributions from the diffuse double layers, ionic adsorption on the hydrophobic side, and the membrane polarization can be assumed to act in an additive manner.

For small surface potentials and weakly polarizable membranes, the contribution from the diffuse double layers to the solid–liquid interfacial energy, Δγ_sl_, is
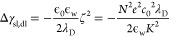
1the contribution from the ionic adsorption is
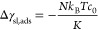
2and the contribution from the membrane to the surface energy is
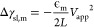
3where *e* is the elementary charge and ζ is the surface potential of the hydrophobic surface. Overall, because we assume a constant surface charge on the hydrophilic side of the membrane and that the hydrophilic side remains wetted even when the hydrophobic side is dewetted, the change in the surface energy of the hydrophobic side due to the charge on the hydrophilic side is typically negligible in the model.

Equations 1 and 3 are direct analogues to the Lipmann equation of electrowetting, where the effective interfacial tension varies with the square of the applied potential. Clearly, there is a direct connection between the polarization across the membrane, as studied here, to the classical electrowetting problem, where the wettability of a droplet on an electrode surface is manipulated by an applied electrode potential.^41^ The experimental findings, in the context of the continuum model, suggest that the electrowetting phenomenon can be extended to membrane systems to control the wettability of the pores.

On the basis of these simplified formulas, we can directly observe that a decrease in the adsorption equilibrium constant, *K*, an increase in the ionic concentration, *c*_0_, and an increase in the applied voltage, *V*_app_, all lead to more wetting of the hydrophobic interface. The observed differences between the iodide and chloride salts may be explained by a difference in the adsorption equilibrium constant at the interfaces, as evidenced by differences in the extent of ionic adsorption of the different ions in MD simulations. On the basis of the model, iodide ions have a smaller equilibrium constant than chloride ions, leading to more adsorption and a greater change in the solid–liquid surface energy for iodide. The trend is fully supported by the nonlinear results in Figure 6b. While the model predictions exhibit a weak dependence on the applied voltage polarity, the model itself does not capture the local concentration changes within the pore, since the concentration within the pore is assumed to be voltage independent. Qualitatively, if we take the concentration changes into account, the model predictions are consistent with the experimental voltage polarity dependence. Because negative voltages in our experimental setup increase local ionic concentrations within the pore, this voltage polarity is more likely to cause wetting of the hydrophobic interface and, in turn, pore wetting.

While the full nonlinear theoretical predictions are qualitatively in agreement with the experimental results, the model cannot yet predict the results in a quantitative manner. It is because large wettability changes (Δγ_sl_ < −10 mJ/m^2^) would require (i) large surface charge density and (ii) large applied voltages. For example, in the absence of applied voltage, the equilibrium constant must be *K* = 0.3 M in order for Δγ_sl_ = −10 mJ/m^2^ at *c*_0_ = 1 M. Benchmarking our theoretical model to our MD simulations, we find that the continuum model (*K* = 0.3 M) corresponds to an adsorbed anion density of 1.1 nm^–2^ at *c*_0_ = 1 M. In molecular simulations of potassium iodide near a hydrophobic interface,^14^ the adsorbed anion density is only 0.2 nm^–2^ at *c*_0_ = 1 M. Using molecular dynamics with electronic continuum correction (ECC) method to capture the anion polarizability more accurately, the adsorbed density in simulations is 0.54 nm^–2^ at *c*_0_ = 1 M.^14^ The continuum model would suggest that in order for the bulk ionic concentration to significantly alter the wettability, the surface charge of the membrane would necessarily be larger than the MD and ECC predictions (shown with stars in Figure 6c). Because the macroscopic contact angle, and thus wettability, was found to be weakly dependent on the bulk ionic concentration (Table S1), the extent of adsorption may be controlled by the nonequilibrium accumulation of anions within and around the pore in response to the applied voltage, instead of the bulk concentration. Nevertheless, in the model, both the increase in concentration and an increase in the applied voltage lead qualitatively to a decrease in the solid–liquid interfacial tension of the hydrophobic side, in agreement with the experiments.

To bring the continuum theoretical predictions closer to the experimentally observed wettability changes, one could include more microscopic details for the bubble, electrolyte, and bipolar membrane system. For example, the bubble and membrane geometry, the nonideal thermodynamics of the electrical double layer, the bubble’s nonequilibrium deformation, and ionic accumulation in the pore could all play a key role in the observed wettability changes. While we have assumed a large blocking bubble that encompasses the pore entrance, the blocking bubbles in the experiments could be as small as the pore size, resulting in more complex electrowetting behavior *within* the pore rather than at the hydrophobic side of the membrane. A more realistic model may require consideration of the contact line pinning at surface heterogeneities, either within or outside the pore, and the effect of ionic charges and electric fields near the pinned contact line, dependent on the precise geometry of the pore/membrane.

For the purposes of our study, we do not extend the continuum theoretical analysis beyond the simple case of the equilibrium wettability of a large blocking bubble in an ideal electrolyte because this limit gives useful trends and physical interpretations without added complexity. Instead, we capture the limit of a small blocking bubble pinned within the pore through the nonequilibrium MD simulation framework, where the applied electric field and concentration changes act to dislodge the bubble, opening the pore to ionic currents as observed in the experiments.

## Conclusions

This article presents an asymmetric hydrophobic nanopore whose transport properties can be gated not only by voltage and type of ion but also by salt concentration. The key to gated transport is the presence of a hydrophobic/hydrophilic junction within the nanopore. Our experimental results clearly indicate that a higher salt concentration facilitates wetting of such asymmetric nanopores. We also show that the effect of salt concentration is linked with voltage dependence of wetting, such that the nanopores are more likely to conduct ion current for the voltage polarity that increases ionic concentrations within the pore. As a result of the voltage dependent wetting, such an asymmetric system functions as a diode for water and all dissolved ions. The mechanism of voltage and salt concentration induced gating was described by using the tools of molecular dynamics as well as a continuum approach of electrowetting. Molecular dynamics simulations predicted local ionic concentrations inside the pore and confirmed accumulation of large iodide ions in the hydrophobic region of the pore that facilitated pore wetting. Simulations also revealed that pore wetting was initiated by short strings of water molecules connecting in the center of the pore. The ionic concentrations obtained from MD simulations were subsequently used as inputs for a continuum model that described salt concentration dependence of wetting by using the framework of electrowetting mechanism. Hydrophobic nanopores gated by salt concentration and voltage could become the basis for nanoscale switches that control transport of water and all species dissolved in it. Such systems could find applications, for example, in drug-delivery systems and ionic circuits.

## Supporting Information Available

Additional experimental recordings, molecular dynamics simulations, and details of the continuum model (PDF)
