# Supplementary material for: Rectified and Salt Concentration Dependent Wetting of Hydrophobic Nanopores
Source: J Am Chem Soc. 2022 Jun 21;144(26):11693–705. doi: 10.1021/jacs.2c03436 (PMC9264351; doi:10.1021/jacs.2c03436)
Supplement: Supplementary file 1 — ja2c03436_si_001.pdf [file ja2c03436_si_001.pdf]

Supporting Information

Rectified and Salt Concentration Dependent Wetting of Hydrophobic Nanopores

Jake W. Polster,[1,#] Fikret Aydin,[2,#] J. Pedro de Souza,[3,#] Martin Z. Bazant,[3,4] Tuan Anh Pham,[2,*] Zuzanna S. Siwy[1,5,6,*]
[1]*Department of Chemistry, University of California, Irvine, CA 92697, USA*
[2]*Quantum Simulations Group and Laboratory for Energy Applications for the Future, Lawrence Livermore National Laboratory, Livermore, California 94551, USA*
[3]*Department of Chemical Engineering, Massachusetts Institute of Technology, Cambridge, Massachusetts 02139, USA*
[4]*Department of Mathematics, Massachusetts Institute of Technology, Cambridge, Massachusetts 02139, USA*
[5]*Department of Physics and Astronomy,* [6]*Department of Biomedical Engineering, University of California, Irvine, CA 92697, USA*

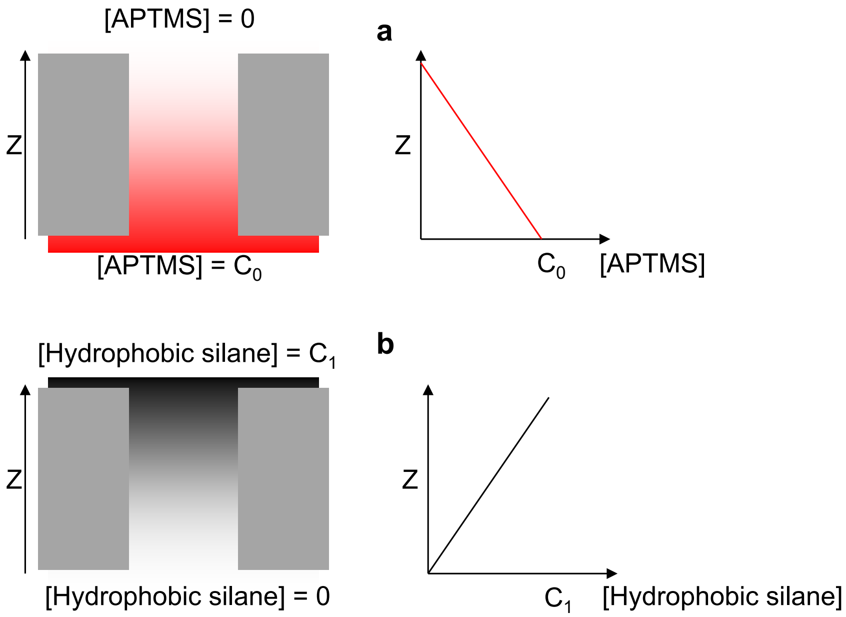

Figure S1. Schemes of two asymmetric modifications used to prepare nanopores with a hydrophobic/hydrophilic junction. The two modifications were performed asymmetrically such that only one side of the pore was in contact with the silane solution, and the other one with a solvent. The shape of the pores is assumed cylindrical, consequently the concentration profiles of the two silanes in the pore are linear.[1] (a) The modification with APTMS was done first, with the APTMS solution placed at the bottom of the chip. (b) The second modification using 1*H*,1*H*,2*H*,2*H*-perfluorooctyltrichlorosilane was performed with the hydrophobic silane solution placed on top of the chip.

* Corresponding Authors: zsiwy@uci.edu, Tel. 949-824-8290; pham16@llnl.gov, Tel. 925-423-6501

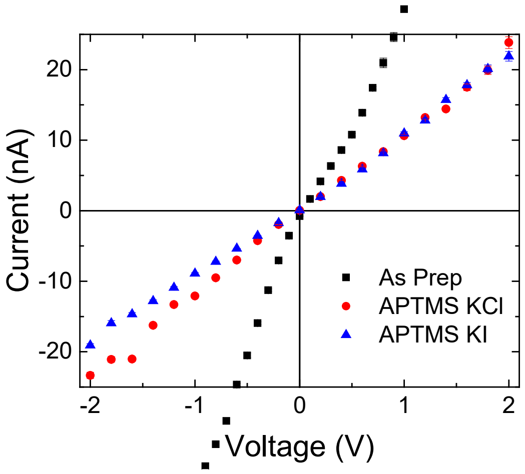

Figure S2. Current-voltage curves of a 14 nm diameter pore as prepared (1 M KCl, black squares), and after symmetric modification with APTMS (1 M KCl in red circles, 1 M KI in blue triangles). The current-voltage curves were obtained by averaging ion current time series recorded at each voltage for 50 s. The error bars are standard deviations of current, also calculated from the time series data. The recordings allowed us to estimate the thickness of the attached APTMS to 3 nm.

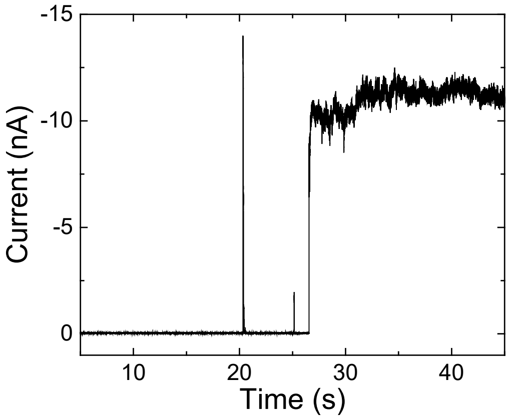

Figure S3. Recording of ion current time series for the pore shown in Figure 3d for 500 mM KI at -1.8 V. This time series was recorded directly after the data in Figure 2 in the main manuscript was acquired. The pore opening probability for this voltage was 0.53.

Table S1. Measurements of contact angle on flat silicon nitride surface before and after modification with 1*H*,1*H*,2*H*,2*H*-perfluorooctyltrichlorosilane. We estimate 10% error based on image processing for all angles.

Salt	Concentration (mM)	Clean SiN_x contact angle (deg)	Hydrophobically modified SiN_x contact angle (deg)
H_2O	-	62	110
KCl	1	72	120
	10	55	110
	100	68	111
	500	65	117
	1000	75	110
	2000	74	114
KI	1	67	117
	10	60	113
	100	61	103
	500	67	103
	1000	65	110
	2000	63	113

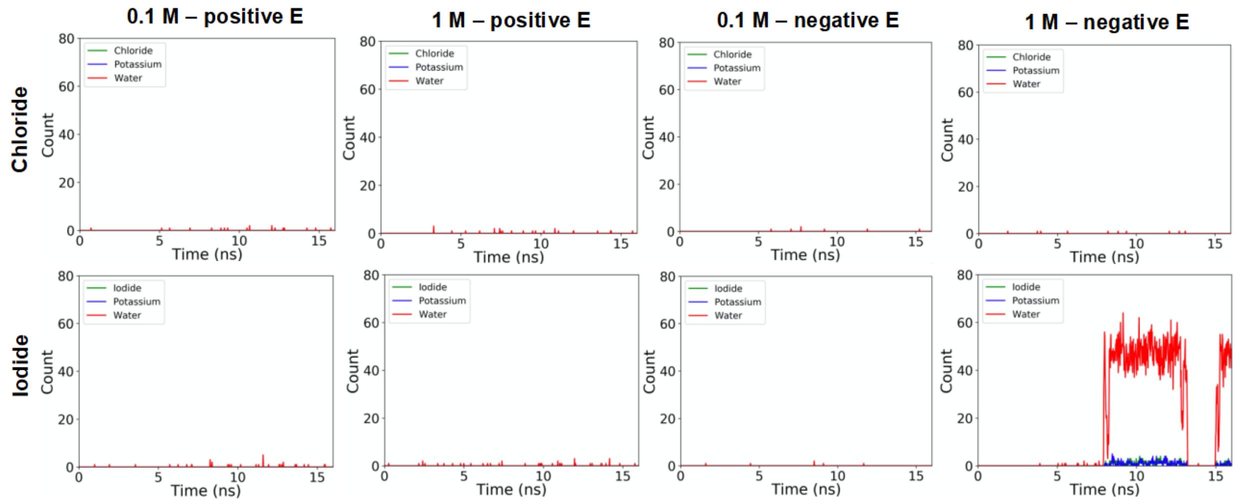

Figure S4. Molecular dynamics simulations of the number of water molecules and ions inside the hydrophobic region of the pore as a function of time at different conditions (low and high concentration, ion type, different polarities of electric fields). The magnitude of electric field is 0.008 V/Å at all conditions. The length of the hydrophobic region chosen for this analysis is 0.6 nm.

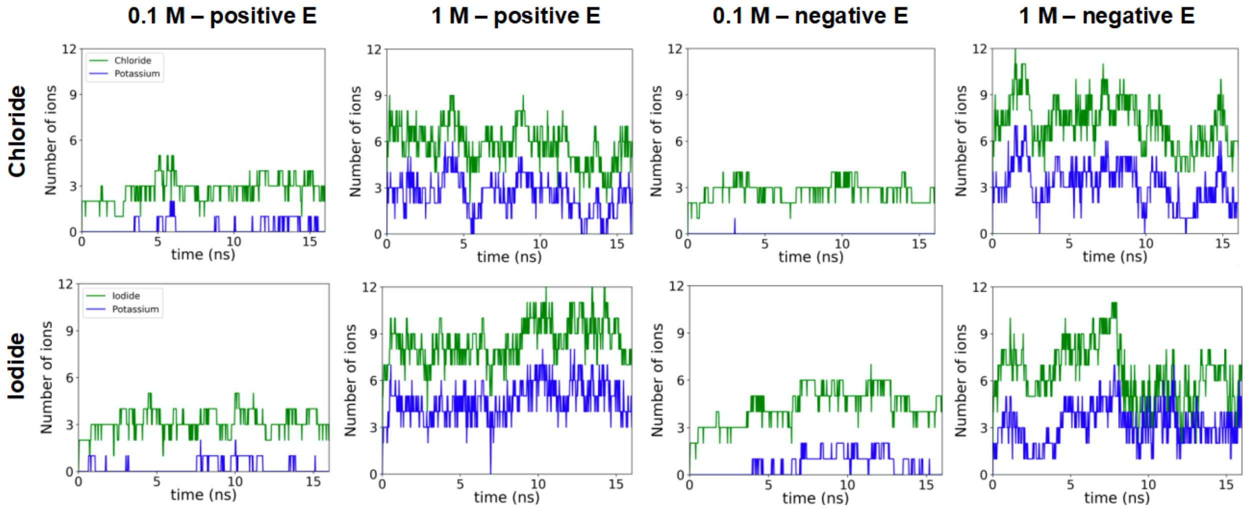

Figure S5. Number of ions inside the hydrophilic region of the pore as a function of time at different conditions (low and high concentration, ion type, different polarities of electric fields). The magnitude of electric field is 0.008 V/Å at all conditions. The length of the hydrophilic region chosen for this analysis is 1.5 nm.

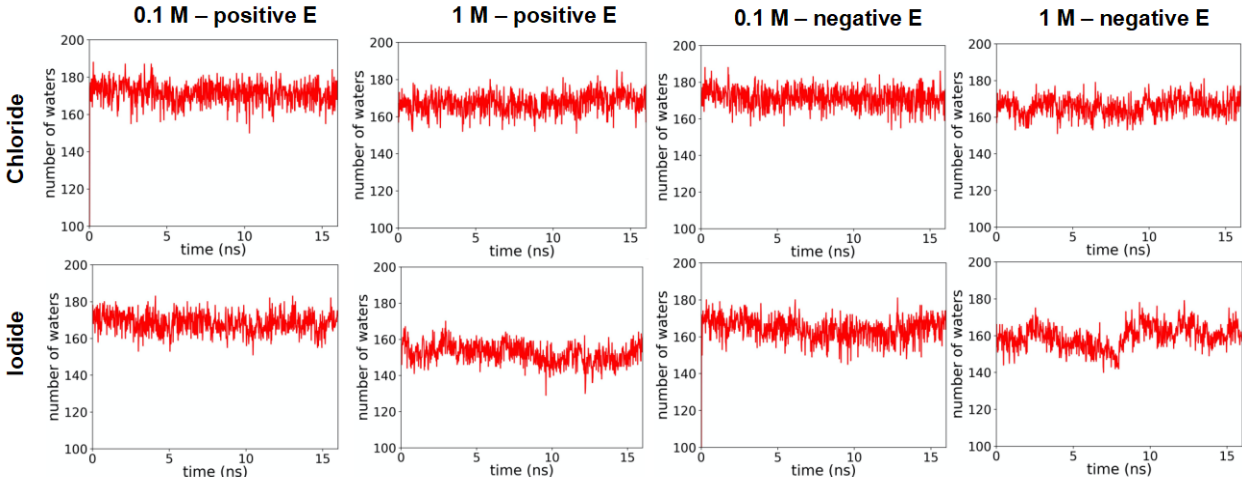

Figure S6. Number of water molecules inside the hydrophilic region of the pore as a function of time at different conditions (low and high concentration, ion type, different polarities of electric fields). The magnitude of electric field is 0.008 V/Å at all conditions. The length of the hydrophilic region chosen for this analysis is 1.5 nm.

Table S2. Average number of ions near the water-vacuum interface in the pore before the pore is open. Results from molecular dynamics simulations.

	Negative E (1M)	Positive E (1M)
Chloride	0.64 ± 0.76	0.50 ± 0.64
Iodide	1.83 ± 1.19	1.29 ± 0.98

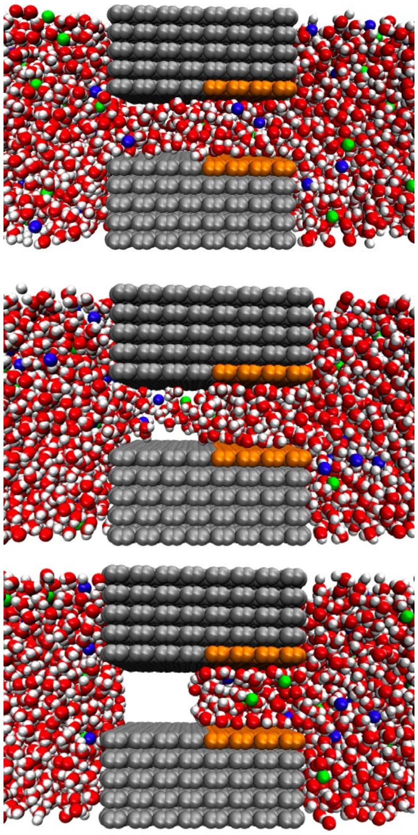

Figure S7. *Simulation snapshots showing different stages of pore dewetting in 1 M KI salt solution at a negative electric field of 0.008 V/Å.*

Theoretical Derivation of Continuum Electrowetting Model

Defining model equations

As sketched in Figure 6a, the electrowetting model is based on computing the electrostatic potential profiles in the electrical double layers at the membrane electrolyte interfaces and in the membrane domain. In regions of the membrane where the hydrophobic side is dewetted (where a gas bubble is in contact with the hydrophobic side), the double layer is only present on the hydrophilic region to screen the surface charge of the hydrophilic side. On the other hand, in regions where the hydrophobic side is in contact with electrolyte solution, the double layers are present on both sides.

Furthermore, when electrolyte is in contact with both sides of the membrane, the applied electrostatic field can polarize the membrane domain, impacting the electrostatic energy of the interface.

Here, in the electrolyte domains that are in contact with solid surfaces, we solve Poisson's equation:

$$\epsilon_w \frac{d^2\phi}{dx^2} = -\rho = \sum_i z_i F c_i \quad \begin{cases} x < -L, & \text{if hydrophobic side dewetted} \\ x < -L \text{ and } x > 0, & \text{if hydrophobic side wetted} \end{cases}. \tag{S1}$$

Here, ϵ_w is the dielectric constant of water, ϕ is the electrostatic potential, x is the spatial coordinate normal to the membrane surfaces, ρ is the volumetric charge density, z_i is the valency of ion i, F is the Faraday constant, c_i is the concentration of the species i, and L is the thickness of the membrane. In the membrane domains, we solve Laplace's equation (assuming the membrane is an ideal dielectric medium with constant membrane dielectric constant, ϵ_m).

$$\frac{d^2\phi}{dx^2} = 0, \quad -L \leq x \leq 0. \tag{S2}$$

In each domain, we have assumed that the field only varies in the normal direction, valid far away from the bubble contact line and from the pore mouth opening.

Next, we outline the strategy to solve for the electrostatic and chemical energy in the double layers and membrane domain in order to predict the effective changes in the solid-liquid interfacial tension, $\Delta\gamma_{sl}$.

Following Genet et al.[2] we assume that the double layers are in equilibrium with the bulk solutions of fixed concentration, with ideal thermodynamics. On the hydrophilic side, the concentrations follow a Boltzmann distribution:

$$c_i = c_{i0} \exp\left(-\frac{z_i F(\phi - V_{\text{app}})}{RT}\right), \quad x < -L \tag{S3}$$

and on the hydrophobic side, they follow a similar Boltzmann distribution with the bulk solution when the hydrophobic side is wetted:

$$c_i = c_{i0} \exp\left(-\frac{z_i F\phi}{RT}\right), \quad x > 0. \tag{S4}$$

Here, c_{i0} is the bulk concentration of species i (equal to the bulk salt concentration, c_0, for the symmetric 1:1 solutions investigated here), R is the gas constant, and T is the absolute temperature. Such a double layer equilibrium assumption is only valid far from the pore opening, where the double layers are unperturbed by the applied currents. Realistically, the concentrations can be significantly perturbed from their bulk values near and within the pore mouth, but we neglect such effects in order to reduce the complexity of the model.

At the membrane interfaces, we enforce boundary conditions based on the amount of adsorbed surface charge. When the hydrophobic side is dewetted, the only boundary condition is:

$$\epsilon_w \frac{d\phi_w}{dx}(x = -L) = q_{s,\mathrm{phil}}. \tag{S5}$$

Since the field is not considered within the membrane domain when the hydrophobic side is dewetted. When the hydrophobic side is wetted, the boundary conditions at each interface are given by:

$$-\epsilon_m \frac{d\phi_m}{dx}(x = -L) + \epsilon_w \frac{d\phi_w}{dx}(x = -L) = q_{s,\mathrm{phil}} \tag{S6}$$

$$\epsilon_m \frac{d\phi_m}{dx}(x = 0) - \epsilon_w \frac{d\phi_w}{dx}(x = 0) = q_{s,\mathrm{phob}}. \tag{S7}$$

The w subscript means that the electric field is evaluated on the electrolyte side of the interface, while the m subscript means that the electric field is evaluated on the membrane side of the interface. The surface charge per unit area on the hydrophilic side is $q_{s,\mathrm{phil}}$, while that on the hydrophobic side is $q_{s,\mathrm{phob}}$. Far away, the potential must match the applied potential in the bulk:

$$\phi(-\infty) = V_{\mathrm{app}} \tag{S8}$$

$$\phi(\infty) = 0. \tag{S9}$$

While we assume the amount of surface charge is fixed on the hydrophilic side, we assume that the amount of surface charge on the hydrophobic side is dependent on the local concentration of anion (either iodide or chloride) that adsorbs to the hydrophobic interface—a charge regulation boundary condition:

$$q_{s,\mathrm{phob}} = -e\,N\,\alpha = -\frac{e\,Nc_0 \exp\left[\frac{\mathrm{F}\phi(\mathrm{x=0})}{\mathrm{RT}}\right]}{c_0 \exp\left[\frac{\mathrm{F}\phi(\mathrm{x=0})}{\mathrm{RT}}\right] + K} \tag{S10}$$

In the above formula, e is the elementary charge, N is the number density of surface sites where anions can adsorb, α is the fraction of surface sites on which anions are adsorbed, and K is the equilibrium constant of anionic adsorption. At large concentrations of anions relative to the equilibrium constant, the surface charge saturates to a constant set by the number of surface sites:

$$q_{s,\mathrm{phob}} = -e\,N. \tag{S11}$$

For small potentials and adsorbed charge fractions, the surface charge is approximated by:

$$q_{s,\mathrm{phob}} = -\frac{eNc_0}{K}. \tag{S12}$$

We will return to the approximate values of the surface charge when approximating the contribution to the energy of the double layer in linear response.

While the system of differential equations is nonlinear and difficult to solve exactly, we can perform one analytical integration to derive algebraic equations relating the potential at the membrane interfaces to the surface charge densities:[2]

$$\left(q_{s,\mathrm{phil}} - Q\right)^2 = 4\epsilon_W RT c_0 \left(\cosh\left(\frac{\mathrm{F}\phi(\mathrm{x}=0)}{\mathrm{RT}}\right) - 1\right) \quad \text{(S13)}$$

$$\left(q_{s,\mathrm{phob}} + Q\right)^2 = 4\epsilon_W RT c_0 \left(\cosh\left(\frac{\mathrm{F}(\phi(\mathrm{x}=-\mathrm{L})-\mathrm{V_{app}})}{\mathrm{RT}}\right) - 1\right) \quad \text{(S14)}$$

$$Q = \frac{\epsilon_m}{L}\left(\phi(x=-L\,) - \phi(x=0)\right) \quad \text{(S15)}$$

These three equations (S13, S14, and S15) with an additional substitution from equation S10 can be solved simultaneously for the three unknowns: $\phi(x=0), \phi(x=-L),$ and Q. The variable *Q* signifies the extent of polarization of the membrane domain, and controls the coupling between the different sides of the membrane domain. When the hydrophobic side is dewetted, *Q*=0. As we will see in the following subsection, we can express the free energy of the double layers and membrane domain in terms of these unknowns, so we can reduce the complexity of the problem down to solving these three algebraic equations.

Defining the surface energy changes

So far, we have outlined the complete set of equations that can be systematically solved as a function of the membrane/electrolyte parameters to find the electrostatic potential at the membrane interfaces and the fraction of sites with adsorbed anions on the hydrophobic side. Now, we need to synthesize the information from the electrostatic model into a calculation of the free energy change due to anion adsorption, membrane polarization, and the free energy stored in the electrical double layers, where we will follow Ref. [[3]] with modifications to account for membrane polarization. From these free energy contributions, we can then make a prediction for the shift in the solid-liquid surface energy.

First, the chemical energy due to anion adsorption is given by:

$$F_{\mathrm{ads}} = \mathrm{N}\mathrm{k}_B T \ln(1-\alpha), \quad \text{(S16)}$$

where k_B is the Boltzmann constant. The free energy of the double layer on the hydrophobic side is:

$$F_{dl,\mathrm{phob}} = -16RT c_0 \lambda_D \sinh^2\left(\frac{F\,\phi(x=0)}{4RT}\right), \quad \text{(S17)}$$

and the free energy on the hydrophilic side is:

$$F_{dl,\mathrm{phil}} = -16RT c_0 \lambda_D \sinh^2\left(\frac{F\,\left(\phi(x=-L)-V_{\mathrm{app}}\right)}{4RT}\right). \quad \text{(S18)}$$

In the above equations, the parameter λ_D is the Debye length. The free energy of the membrane domain is:

$$F_{\mathrm{m}} = -\frac{\epsilon_m}{2L}\left(\phi(x=0) - \phi(x=-L)\right)^2. \quad \text{(S19)}$$

Now, we can synthesize the different free energy contributions of the double layer to predict the change in the apparent solid-liquid surface energy. Here, we assume that $\gamma_{sl}^{\text{base}}$ is the solid-liquid surface tension of the hydrophobic side in the absence of applied voltage or anion adsorption. We calculate the shift in the apparent solid liquid surface tension on the hydrophobic side when there is nonzero membrane polarization and anion adsorption, $\Delta\gamma_{sl}$:

$$\gamma_{sl,\text{phob}} = \gamma_{sl}^{\text{base}} + \Delta\gamma_{sl} \ . \tag{S20}$$

The quantity $\Delta\gamma_{sl}$ can be further decomposed into contributions from (i) the ionic adsorption, $\Delta\gamma_{sl,\text{ads}}$ (ii) the double layers, $\Delta\gamma_{sl,\text{dl}}$, and (iii) the membrane polarization $\Delta\gamma_{sl,\text{m}}$.

From the definitions of the free energy, the ionic adsorption contribution is:

$$\Delta\gamma_{sl,\text{ads}} = F_{\text{ads}} \tag{S21}$$

The contribution from the double layers is:

$$\Delta\gamma_{sl,dl} = F_{dl,\text{phob}}^{\text{wetted}} + F_{dl,\text{phil}}^{\text{wetted}} - F_{dl,\text{phil}}^{\text{dewetted}} \ , \tag{S22}$$

where we have set the reference free energy of the hydrophilic side to the free energy of the hydrophilic side with the hydrophobic side in the dewetted state. The contribution from the membrane polarization is:

$$\Delta\gamma_{sl,\text{m}} = F_{\text{m}}. \tag{S23}$$

In generating Figure 6b-c, these contributions are computed numerically from the nonlinear equations and summed together to give $\Delta\gamma_{sl}$.

Approximating the contributions to solid-liquid interfacial energy
As explained in the main text, we can generate useful formulas by assuming small potentials, low surface adsorption, and a weakly polarizable membrane. While these formulas do not accurately describe the full nonlinear solutions, they display the same qualitative trends, and allow for quick computations.

First, for anionic adsorption, starting from equation S16, the chemical energy due to adsorption for small values of α is:

$$\Delta\gamma_{sl,ads} = -Nk_BT\alpha = -\frac{N\,k_BT\,c_0}{K}. \tag{S24}$$

For the membrane domains, if there are only small perturbations in potential due to the double layers, then the membrane polarization is mainly attributable to the applied voltage:

$$\Delta\gamma_{sl,m} = -\frac{\epsilon_m}{2L}V_{\mathrm{app.}}^2 \quad \text{(S25)}$$

Finally, the double layer contribution from the hydrophilic side of the membrane will be zero under the simplifying assumptions, since due to the low polarizability of the membrane and the constant surface charge of the hydrophilic side, the double layer contribution from the hydrophilic side is identical whether the hydrophobic side is wetted or dewetted. The only contribution from the double layer will come from the hydrophobic side. There, the surface potential can be approximated as:

$$\phi(x = 0) = \zeta \approx \frac{-Nec_0\lambda_D}{K\epsilon_w}, \quad \text{(S26)}$$

And assuming small potentials, the double layer contribution can be approximated from the expanded form of equation S17 as:

$$\Delta\gamma_{sl,dl} = -\frac{\epsilon_w}{2\lambda_D}\zeta^2 = -\frac{N^2e^2c_0^2\lambda_D}{2\epsilon_w K^2}. \quad \text{(S27)}$$

References

1. Berg, H. C., *Random Walks in Biology*. Princeton University Press: 1993.
2. Genet, S.; Costalat, R.; Burger, J., A Few Comments on Electrostatic Interactions in Cell Physiology. *Acta Biotheoretica* **2000,** *48* (3), 273-287.
3. Virga, E.; Spruijt, E.; de Vos, W. M.; Biesheuvel, P. M., Wettability of Amphoteric Surfaces: The Effect of pH and Ionic Strength on Surface Ionization and Wetting. *Langmuir* **2018,** *34* (50), 15174-15180.
